# Supplementary material for: Identification of photoperiod-induced specific miRNAs in the adrenal glands of Sunite sheep (Ovis aries)
Source: Front Vet Sci. 2022 Jul 22;9:888207. doi: 10.3389/fvets.2022.888207 (PMC9354845; doi:10.3389/fvets.2022.888207)
Supplement: Supplementary file 1 [file Data_Sheet_1.ZIP › Supplementary file/miRDeep2 file.zip!/miRDeep2 file/Know_Structure/SPLP42A2/oar-mir-431.pdf]

miRBase precursor	: oar-mir-431
Total read count	: 258
oar-miR-431 read count	: 36
remaining reads	: 222

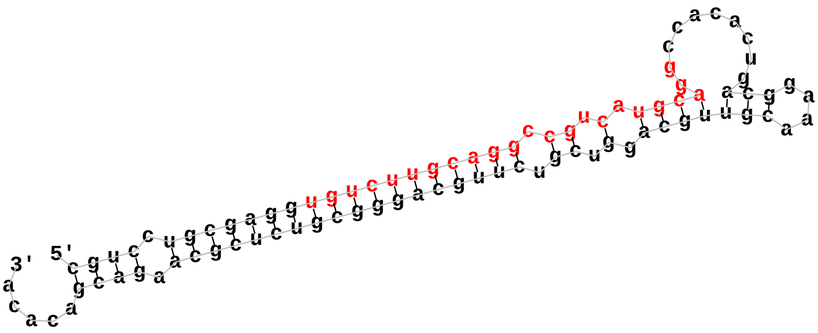

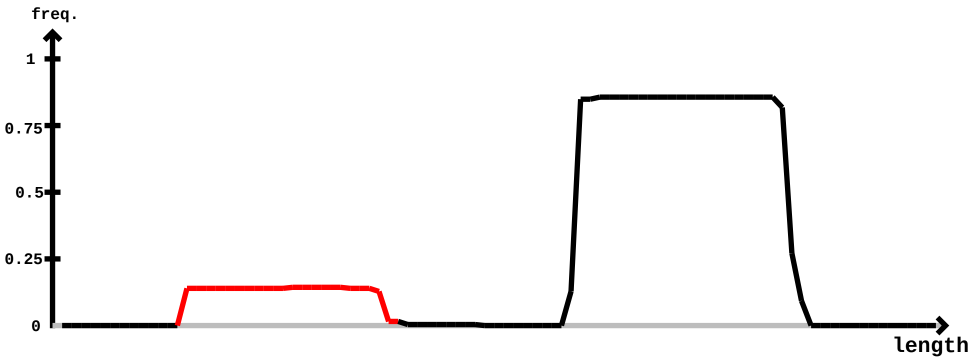

oar-miR-431

```
5'- cguccugcgaggugucuugcaggccgucaugcaggccacacugacggaaacguugcaggucgucuugcagggcgucucgcaagacgacaca   -3'    exp
    ((((.((((((((((((((((((.((.(.((((..........(((....))))))).).)).)))))))))))))))))))).)))).....    reads      mm         sample
    ............ugucuugcaggccguca..............................................................    1          0          seq
    ............ugucuugcaggccgucaugc...........................................................    3          0          seq
    ............ugucuugcaggccgucaugca..........................................................    29         0          seq
    ............ugucuugcaggccgucaugcagg........................................................    3          0          seq
    .......................ccgucaugcaggccacacug................................................    1          0          seq
    ....................................................uugcaggucgucuugcagggcg.................    2          0          seq
    ....................................................uugcaggucgucuugcagggcgu................    25         0          seq
    ....................................................uugcaggucgucuugcagggcguc...............    3          0          seq
    ....................................................uugcaggucgucuugcagggcgucu..............    3          0          seq
    .....................................................ugcaggucgucuugcagggcg.................    7          0          seq
    .....................................................ugcaggucgucuugcagggcgu................    115        0          seq
    .....................................................ugcaggucgucuugcagggcguc...............    43         0          seq
    .....................................................ugcaggucgucuugcagggcgucu..............    21         0          seq
    .......................................................caggucgucuugcagggcg.................    1          0          seq
    .......................................................caggucgucuugcagggcgu................    1          0          seq
```
